# The Short Non-Coding Transcriptome of the Protozoan Parasite *Trypanosoma cruzi*

**DOI:** 10.1371/journal.pntd.0001283

**Published:** 2011-08-30

**Authors:** Oscar Franzén, Erik Arner, Marcela Ferella, Daniel Nilsson, Patricia Respuela, Piero Carninci, Yoshihide Hayashizaki, Lena Åslund, Björn Andersson, Carsten O. Daub

**Affiliations:** 1 Science for Life Laboratory, Department of Cell and Molecular Biology, Karolinska Institutet, Stockholm, Sweden; 2 RIKEN Omics Sciences Center, RIKEN Yokohama Institute, Yokohama, Kanagawa, Japan; 3 Rudbeck Laboratory, Department of Immunology, Genetics and Pathology, Uppsala University, Uppsala, Sweden; 4 Institute of Cell Biology, University of Bern, Bern, Switzerland; International Centre of Insect Physiology and Ecology, Kenya

## Abstract

The pathway for RNA interference is widespread in metazoans and participates in numerous cellular tasks, from gene silencing to chromatin remodeling and protection against retrotransposition. The unicellular eukaryote *Trypanosoma cruzi* is missing the canonical RNAi pathway and is unable to induce RNAi-related processes. To further understand alternative RNA pathways operating in this organism, we have performed deep sequencing and genome-wide analyses of a size-fractioned cDNA library (16–61 nt) from the epimastigote life stage. Deep sequencing generated 582,243 short sequences of which 91% could be aligned with the genome sequence. About 95–98% of the aligned data (depending on the haplotype) corresponded to small RNAs derived from tRNAs, rRNAs, snRNAs and snoRNAs. The largest class consisted of tRNA-derived small RNAs which primarily originated from the 3′ end of tRNAs, followed by small RNAs derived from rRNA. The remaining sequences revealed the presence of 92 novel transcribed loci, of which 79 did not show homology to known RNA classes.

## Author Summary

Chagas' disease is a major health problem in Latin America and is caused by the protozoan parasite *Trypanosoma cruzi*. *T. cruzi* lacks the pathway for RNA interference, which is widespread among eukaryotes, and is therefore unable to induce RNAi-related processes. In many organisms, small RNAs play an important role in regulating gene expression and other cellular processes. In order to understand if other small RNA pathways are operating in this organism, we performed high throughput sequencing and genome-wide analyses of the short transcriptome. We identified an abundance of small RNAs derived from non-coding RNA genes, including transfer RNAs, ribosomal RNAs as well as small nucleolar RNAs and small nuclear RNAs. Certain tRNA types were overrepresented as precursors for small RNAs. Further, we identified 79 novel small non-coding RNAs, not previously reported. We did not identify canonical small RNAs, like microRNAs and small interfering RNAs, and concluded that these do not exist in *T. cruzi*. This study has provided insights into the short transcriptome of a major human pathogen and provided starting points for further functional investigation of small RNAs and their biological roles.

## Introduction

*Trypanosoma cruzi* is a protozoan parasite and the causative agent of Chagas' disease, which has substantial health and socioeconomic impact in Latin America [1]. Treatment is currently restricted to a small number of drugs with insufficient efficacy and potentially harmful side effects.

The genome of *T. cruzi* strain CL Brener is complex in terms of sequence repetitiveness and is a hybrid between two diverged haplotypes, named non-Esmeraldo-like and Esmeraldo-like: we refer to them here as non-Esmeraldo and Esmeraldo. Taken together, both haplotypes [2] sum up to approximately 110 Mb distributed over at least 80 chromosomes [3]. Similar to other trypanosomatids, genes are organized into co-directional clusters that undergo polycistronic transcription. Gene rich regions are frequently interrupted by sequence repeats, which comprise at least 50% of the genome [2]. Several gene variants occur in tandemly repeated copies which often collapse in shotgun assemblies [4]. The genome of a different, non-hybrid strain named Sylvio X10 was recently sequenced and partially assembled, showing a core gene content highly similar to CL Brener [5].

The *T. cruzi* life cycle is complex and consists of several distinct life stages, morphological states and hosts [1]. To achieve successful completion of the life cycle, the parasite must rapidly adapt to different environments by regulating its gene expression [6]. Transcription in *T. cruzi* often, but not exclusively, starts at strand switch regions [7], [8], where long transcripts are produced by RNA polymerase II and matured via *trans*-splicing and polyadenylation [9]. There is to date no definite model of how and if transcription is regulated, as RNA polymerase II promoters for protein-coding genes have not been identified. Thus, it is thought that gene expression is mainly regulated at the post-transcriptional level [9].

RNA interference (RNAi) and related pathways are widespread in animals and other metazoans, participating in a wide range of cellular processes; from chromatin organization to silencing of genes and selfish elements. RNAi relies on small RNA molecules, approximately 20–30 nucleotides in length, to trigger target silencing. In eukaryotes, several different types of small RNAs have been identified. Of these, the best characterized are microRNAs (miRNAs) and small interfering RNAs (siRNAs). See Table 1 for a summary of small RNAs discussed in this study. Two proteins are required for small RNA biogenesis and function: Dicer and Argonaute. Among protozoan parasites, the RNAi machinery has either been lost or retained. *T. cruzi* have lost the canonical RNAi machinery, which has been confirmed both functionally [10] and from the genome sequence [2], although RNAi is functional in certain other trypanosomatid species [11], [12], [13]. In the African trypanosome *Trypanosoma brucei*, convincing evidence has shown the presence of an active RNAi machinery (see [14] for references) and more recently pseudogene-derived small RNAs, which were reported to suppress gene expression [15]. A similar situation has been observed in the Leishmania genus. *Leishmania braziliensis* possesses a functional RNAi-pathway [16], whereas other members of this genus do not [12]. Analyses of the *T. cruzi* genome have revealed lack of both Dicer and Argonaute homologs. However, similar to other trypanosomatids, *T. cruzi* possess a protein with a solo Piwi domain, but without a PAZ domain. The biological role of this protein is presently unknown, although it has been suggested to represent a member of a novel Argonaute subfamily [17].

**Table 1:** Classes of non-coding RNAs discussed in this paper.

Short name of RNA classes	Full name of RNA classes	Notes	References
ncRNAs	Non-coding RNAs	Generic term for non-protein coding RNAs	[22], [42], [66]
sncRNAs	Small non-coding RNAs	Generic term for small non-protein coding RNAs	[22], [42]
piRNAs	Piwi interacting RNAs	Involved in retrotransposon silencing	[67]
miRNAs	microRNAs	21-24 nucleotides in length and involved in regulation of gene expression	[68]
snoRNAs	Small nucleolar RNAs	Guide chemical modifications of other non-coding RNAs	[52], [69]
snRNAs	Small nuclear RNAs	Involved in various processes such as RNA splicing	[69]
siRNAs	Small interfering RNAs	Double stranded RNAs that act in various silencing pathways	[70]
tsRNAs	tRNA-derived small RNAs	Small RNAs derived from tRNAs	[37]

To date, little is known about the presence of small non-coding RNAs (sncRNAs) in trypanosomatids, which do not depend on RNAi. Recently, one study reported the prediction of sncRNAs in the trypanosomatids [18], providing evidence of yet uncharacterized sncRNAs in these species. However, comparative genomics suffers from the limitation that it does not facilitate identification of species-specific small RNAs or regulatory elements. Furthermore, another study described small-scale sequencing of small RNAs in *T. cruzi*, and reported a population of tRNA-derived small RNAs, which was linked to cellular stress [19]. Moreover, studies from another unusual eukaryote, *Giardia lamblia,* have shown that sncRNA can be highly diverged from metazoan sncRNA [20], [21] and therefore escape detection using homology searches. Lessons from non-protozoans have taught that novel sncRNAs are often in low abundance and avoid detection using conventional techniques [22], which do not sequence deep enough to capture the full complexity of the transcriptome.

In order to obtain a more complete picture of the short *T. cruzi* transcriptome, we have performed unbiased deep sequencing and genome wide analyses of the short transcriptome from *T. cruzi* epimastigotes. The data indicated the existence of an abundance of small RNAs derived from non-coding RNAs and a number of novel expressed loci in the genome.

## Materials and Methods

### Sequence data

The sequences have been deposited in the DNA Data Bank of Japan under the accession number DRA000396 and are also available for download from http://www.ki.se/chagasepinet/ncrna.html.

### Cell culture, library preparation and sequencing

Epimastigotes from *T. cruzi* CL Brener were grown exponentially at 28°C in liver infusion tryptose (LIT) media [23] supplemented with 10% FBS (Gibco) and streptomycin/penicillin (Gibco), pH 7.3. Total RNA was extracted using the TRIzol method (TRI Reagent, Sigma) following manufacturers' instructions. The total RNA was converted to cDNA using a standard protocol and size fractioned using a polyacrylamide gel. The sequencing library was generated according to the manufacturers' instructions and sequenced with a 454 instrument (GS20 FLX).

### Bioinformatics analyses

The sequence data was stripped of the 3′ ‘CCA’ extension and aligned with the *T. cruzi* genome assembly [3] using the Burrows-Wheeler Aligner (BWA) [24]. BWA was configured to allow up to two mismatches. Repetitive elements were identified using RepeatMasker [25] and RepBase [26] (r16.01). Only repeats longer than 40 bp were considered.

Identification of novel expressed genomic loci was performed using clustering of reads which could not otherwise be assigned an identity. Clustering was done on reads satisfying the following criteria: i) they do not have an annotation (i.e. tRNA, etc); ii) have only one valid alignment in the genome (single mapping); iii) have an overlap of at least one base with another read. Subsequently, the resulting clusters were filtered using the following criteria: i) a cluster should contain at least six reads ii) at least two reads should be unique. The resulting novel non-coding RNAs were manually examined and assigned putative identities using BLAST.

A sequence database of trypanosomatid genes was established by extracting sequences containing ‘trypanosoma’ in the header line from the GenBank non-redundant database. Statistical evaluation and charts were performed using the R platform. Homology searches were done using NCBI BLAST.

Prediction of tRNA secondary structure was performed using tRNAScan-SE [27] and visualized using VARNA [28]. Prediction of secondary structures of novel small RNAs was done with Vienna RNA [29]. Analysis of putative microRNA targets was performed using TargetScan [30] and GoTermMapper [31]. Scripts were written in Perl and are available on request.

### Stem-loop real time PCR

Stem-loop real time-PCR experiments and primer design were performed as described in [32]. The quality of RNA samples was assessed on an 1.5% TBE-agarose gel. All RNA samples were treated with DNAse I (Fermentas) previous to the reverse transcriptase reaction. SnoRNA or 5S rRNA was used as reference RNA for qualitative/quantitative experiments. The following reagents were mixed and subjected to Pulsed RT reaction; 60 ng of *T. cruzi* RNA (per RT reaction), 0.5 mM dNTP, 10X First Strand Buffer, 5 mM MgCl2, 10 mM DTT, RNAseOUT, 50 units Superscript III RT (Invitrogen) and 1 µl of SL-RT specific primer, using the conditions: 30 min at 16°C followed by 60 cycles of 30 s at 30°C, 30 s at 42°C, 1 s at 50°C and a final step of 5 min at 85°C. RNAse H was added and incubated for 20 min at 37°C. The real time PCR reactions were performed in triplicates using 1 µl cDNA, 300 nM forward specific and reverse universal primers and 2X SYBR Green Master Mix (Roche). Cycling conditions: 5 min at 95°C, 40 cycles 95°C-5 s, 60°C-20 s, 72°C-1 s followed by dissociation curve analysis in Strategene Thermal Cycler Mx3000P. Experiments were repeated at least twice per each of two biological samples. Ct values were normalized against 5S rRNA values and the abundance ratio was calculated for each individual Ct value and mean as well as standard deviation were calculated and graphed using SigmaPlot 9.0. The 5S rRNA (Tc00.1047053509455.160) and C/D snoRNA (Tc00.1047053510739.50) were used for normalization. Primers used are listed in Table S3.

## Results and Discussion

### Library characteristics and mapping

**Figure 1:**
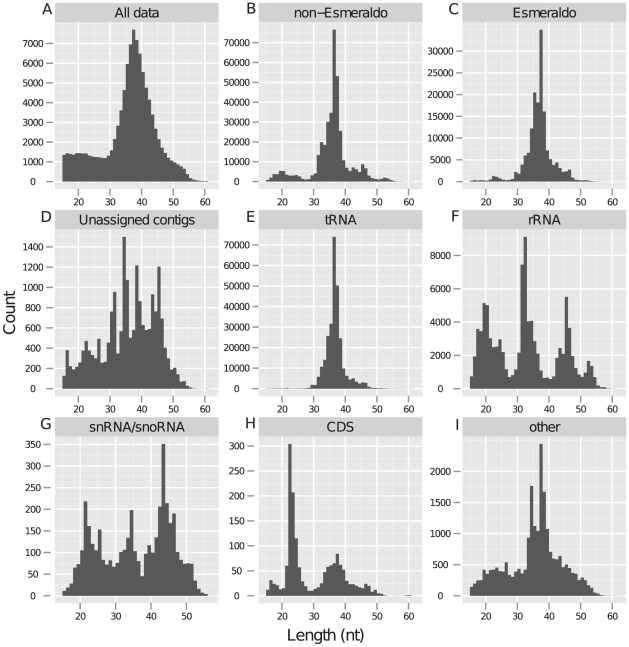
Length distribution of sequenced small RNAs. Histograms show the length distribution of sequenced small RNAs. A) The total sequenced data. B) Small RNAs aligned with non-Esmeraldo. C) Small RNAs aligned with Esmeraldo. D) Small RNAs aligned with unassigned contigs. E) Small RNAs derived from tRNA. F) Small RNAs derived from rRNA. G) Small RNAs derived from snRNA/snoRNA. H) Small RNAs derived from coding sequences. I) Small RNAs derived from other features than mentioned.

An epimastigote cDNA library was size fractioned on a polyacrylamide gel and sequenced using 454 sequencing [33] (Materials and Methods). Sequencing resulted in a total of 582,243 reads (101,284 unique) with a size range between 16 to 61 nucleotides (Figure 1A). The median sequence length of the library was 38 nt. A total of 12.2% (71,309/582,243) reads occurred as single copy, whereas the remaining reads had a variable copy number between 2 and 41,929. The selected size range should contain only non-coding RNA (ncRNA), as there are no known protein-coding genes in this size range. Further, this size range was selected to avoid spliced leader RNAs. However, degradation products from transcriptional turnover could be present in the sample. Based on two observations we conclude that degradation products were not contaminating the library; i) degradation fragments should exhibit a random distribution pattern in protein-coding genes, which was not the case, ii) ribosomal RNA constitute the bulk (>80%) of cellular RNA, which was not observed in the sequence data.

The sequence data was separately aligned with each of the *T. cruzi* CL Brener haplotypes; non-Esmeraldo and Esmeraldo (Figure 1BC, Materials and Methods). In addition, reads were aligned with a 38 million base pair collection of unassigned contigs (Figure 1D), which mostly consists of repeats [3], [34]. This resulted in a total of 90.7% aligned reads (528,228/582,243), or expressed in unique reads, 74.0% aligned reads (75,024/101,284) (Table 2). Slightly more reads were aligned with non-Esmeraldo, owing to the more complete status of this haplotype assembly compared to Esmeraldo; however the length distribution of the aligned reads were similar (Figure 1BC), indicating both haplotypes might generate similar RNA populations.

**Table 2:** Summary of small RNA alignments.

Mapping class	non-Esmeraldo (# reads) d	Esmeraldo (# reads) d	Unassigned contigs (# reads) d
**Reads aligned** a	501,721 [65,940]	409,757 [54,695]	174,196 [34,914]
**Single mappers** b	388,551 [42,805]	162,622 [24,801]	19,893 [4910]
**Specific** c	24,729 [3689]	1009 [626]	23,040 [7404]

a Total number of reads that can be aligned (multi mappers and single mappers).

b Number of reads with a single alignment location.

c Number of reads that align uniquely to this haplotype/group.

d The number in brackets refers to the number of unique (non-redundant) reads.

A total of 9.2% (53,646/582,243) of the reads could not be aligned with the genome using the default alignment procedure, raising the question if these reads are biologically derived or technical artifacts. The following scenarios are possible; i) unaligned reads are technical artifacts or enriched with sequencing errors, ii) unaligned reads represent small RNAs derived from unfinished parts of the genome sequence, iii) small RNAs have been subjected to chemical modification and RNA editing events. As the *T. cruzi* CL Brener genome sequence is not complete [2], [4] it remains possible that at least some small RNAs are derived from unassembled regions. To investigate this, unaligned reads were mapped to the genomic shotgun reads from the genome project, which provided alignment to 0.49% (2860/53,646) of the unaligned reads. Examination of a limited number of reads, that failed alignment, found homology to tRNA^Lys^. As these reads occurred in a high copy number (∼300) and mismatches were located in the anticodon loop, this makes it possible that mismatches are not sequencing errors but rather modified nucleosides misinterpreted by the sequencer.

### Library composition and content

In order to differentiate between known and unknown RNA species in the library, we categorized reads into classes using genome annotations. Alignment coordinates were superimposed on genome annotations and each read was categorized into one of the categories in Table 3 if it completely or partially overlapped with the annotation. In cases where a tRNA, snRNA or snoRNA was overlapping a protein-coding gene, the ncRNA gene was preferentially selected. To further improve the classification, reads without annotation were queried against a database of various trypanosomatid sequences (Materials and Methods).

**Table 3:** Coverage of genomic features by small RNAs.

RNA class	non-Esmeraldo (# reads) a	Esmeraldo (# reads) a	Unassigned contigs (# reads) a
tRNA	280,417 (72.17%) [28,394]	150,675 (92.65%) [20,445]	2637 (13.26%) [564]
rRNA	94,382 (24.29%) [9994]	3412 (2.1%) [731]	15,506 (77.95%) [3435]
snRNA/snoRNA	3647 (0.94%) [1632]	3193 (1.96%) [1263]	235 (1.18%) [157]
CDS	691 (0.18%) [393]	3011 (1.85%) [896]	22 (0.11%) [10]
mini-exon	0 (0%) [0]	5 (0%) [4]	0 (0%) [0]
other	9414 (2.42%) [2392]	2326 (1.43%) [1462]	1493 (7.51%) [744]
total	388,551 (100%) [42,805]	162,622 (100%) [24,801]	19,893 (100%) [4910]

a Refers to the number of single mapping reads that align either entirely or partially. The number in brackets refers to the number of unique (non-redundant) reads.

For reads with a single alignment location (single mappers), 97.4% (378,446/388,551) of the reads in non-Esmeraldo and 96.7% (157,280/162,622) in Esmeraldo were found to correspond to small ncRNAs (sncRNAs) derived from tRNA, rRNA, snRNA and snoRNA (Table 3). tRNA-derived small RNAs (tsRNA) was found to be the most abundant type in the library, composing at least 65.3% (380,191/582,243) of the total sequence data, which we further describe in the next section. This result suggests that the vast majority of small RNA species in *T. cruzi* epimastigotes are derived from known ncRNA classes.

About 2–5% of the aligned sequences could not be classified into known ncRNA classes. It should be noted that this fraction might not represent the entire abundance of novel sncRNA in *T. cruzi*, as some sncRNA might only be present in a specific life stage or under a certain physiological condition. Furthermore, long novel ncRNAs (>61 nt) could exist [18], as was for example reported in *Leishmania infantum* [35].

A total of 19,893 reads aligned with unassigned contigs, out of which 78% (15,506/19,893) represented reads that aligned with rRNA genes (Table 3). A minor fraction consisted of reads that aligned with tRNA (13%) and snRNA/snoRNA (1%). This is consistent with the fact that few rRNA genes have been properly assembled [2], [3].

### Small RNAs derived from mature transfer-RNAs represent the bulk of the short transcriptome in *T. cruzi*

For both non-Esmeraldo and Esmeraldo, a total of 69.1% (282,036/408,008) of the small RNAs were assigned to the tRNA category (considering single mapping reads), despite the fact that the library was size selected for sequences shorter than 61 nt and mature tRNAs are between 70–80 nt. A closer inspection revealed the presence of tRNA-derived small RNAs (tsRNAs), a phenomenon reported previously in higher eukaryotes [36], [37] and lower eukaryotes [38], [39], [40]. However, the physiological role, if any, of tsRNA is not well defined (for review and discussion see [41], [42], [43], [44]).

*T. cruzi* tsRNAs were first reported by Garcia-Silva et al. [19], who found tsRNA to be recruited to cytoplasmic granules and increase under stress conditions. The authors employed a 20–35 nt cDNA library and sequenced 348 clones, and found that 26% of the clones were derived from tRNA and 60% from rRNA. The study also showed a higher representation for 5′end tRNA derived small RNAs, which may be explained by the relatively low number of clones sequenced in this study.

**Figure 2:**
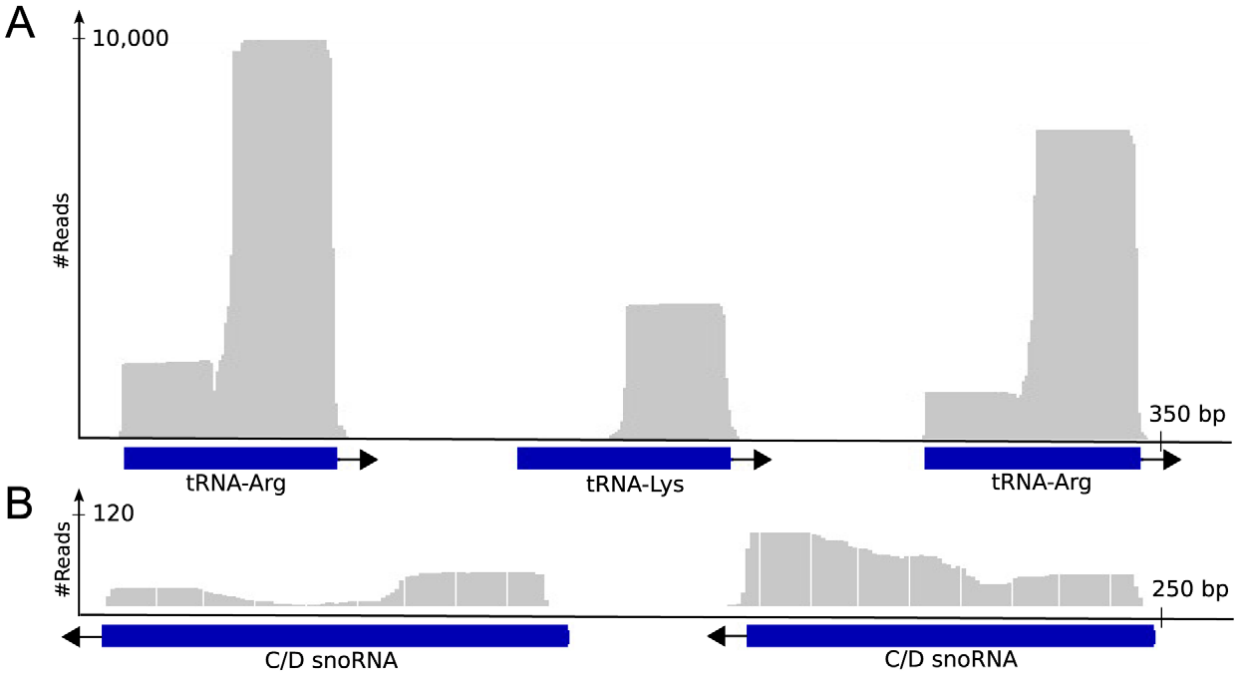
Schematic illustration of small RNA alignments to tRNA and snoRNA genes. Schematic illustration of small RNAs aligned to known non-coding RNA genes (three tRNA genes and two snoRNA genes). The top graphs display the read density along the genes. Blue bars represent genes and arrows indicate the direction of genes (forward or reverse strand). A) Shows three tRNA genes (Tc00.1047053509105.114, Tc00.1047053509105.116, Tc00.1047053509105.118). The 3′ part of the tRNA gene display higher read depth than the 5′ part. B) Shows two C/D small nucleolar genes (Tc00.1047053508461.74, Tc00.1047053508461.75).

In our library, tsRNA had a median length of 38 nt and 88.9% (250,920/282,036) were derived from the 3′ end of tRNAs (Figure 1E, Figure 2, Table 4). Moreover, 75.3% (189,116/250,920) of the 3′-derived reads contained a ‘CCA’ nucleotide extension; indicating that the majority of 3′ tsRNA are derived from mature tRNA species, as the ‘CCA’ addition is post-transcriptionally added in eukaryotes. However, we cannot rule out that the remaining reads did not lose the ‘CCA’ extension during sample or library preparation.

**Table 4:** Small RNAs mapped on tRNAs.

tRNA a	Copies b	% Amino acids c	3′ Reads d	5′ Reads d
His	4	2,45	95,982	3
Arg	12	6,86	40,859	4431
Thr	6	5,99	26,681	2750
Glu	5	6,91	16,151	6888
Ala	5	8,8	15,065	122
Gln	6	3,62	2616	9861
Trp	2	1,2	11,095	43
Ser	7	8,12	10,029	26
Tyr	2	2,36	8481	61
Val	6	7,7	5650	2692
Asp	2	4,83	4885	3156
Gly	8	7,02	4412	338
Lys	6	4,27	3388	47
Pro	6	4,95	1899	13
Ile	6	3,65	1637	34
Leu	11	9,51	745	213
Cys	2	2,15	875	11
Met	6	2,43	468	29
Phe	4	3,63	1	0
Asn	4	3,54	1	0

a Refers to the tRNA type.

b The genomic copy number including both non-Esmeraldo and Esmeraldo.

c The percentage of amino acids in the predicted proteome.

d Refer to the number of single mapping reads that align with annotated tRNA genes.

The median length of 38 nt is consistent with the current view of bisectional cleavage of mature tRNA. Despite this, we also identified shorter tsRNA (<25 nt) albeit in lower frequency; a total of 1605 tsRNA were 24 nt or less and primarily derived from tRNA^Glu^, tRNA^Asp^, tRNA^Tyr^, tRNA^Val^ and tRNA^Arg^ (Figure S1). Interestingly, the shorter tsRNA were more often derived from the 5′ arm. Most tRNA isoacceptors were found to be precursors for tsRNA, but with relative different amounts (Figure S1). The most abundant tsRNA were derived from the 3′ arm of tRNA^His^ and occurred in 41,929 copies and contained the ‘CCA’ extension (Table 5, Figure S2). Interestingly, the 3′/5′ ratio of tsRNA was not equal for all tRNA isoacceptors (Table 4). For example, tRNA^Gln^ showed more tsRNA derived from the 5′ arm.

**Table 5:** Abundantly expressed small RNAs.

Type a	Small RNA b	Frequency c	Location d
tRNA-His (GUG)	TCTGAATACCCGGGTTCGATTCCCGGTCTTCCCTCCA	41,929	3′
rRNA	ATATCGAATCGCCATCCAAATCATCTGGTAGGC	6737	-
rRNA	TCTGCCTGCCCTCGAAGGCGCCAAGTATCTCCATGATCACAAGACA	4316	-
tRNA-Gln (UUG)	GGTCCTATAGTGTAGTGGTTATCACTTCGGACTTT	3063	5′
tRNA-Ala (CGC)	ACGGAAGGCCTAGGGTTCGATCCCCTACTCGTCCACCA	2601	3′
tRNA-Tyr (GUA)	TCACAGGGTCGCTGGTTCGTTTCCGGCCGGAAGGACCA	2332	3′
tRNA-Trp (CCA)	TCCAGGGGTCGCAGGTTCAATCCCTGCAGTCCTCACCA	1992	3′
rRNA	TCTGCCTGCCCTCGAAGGCGCCAAGTATCTCCATGATCACAAGACAT	1951	-
rRNA	ACACGTCCCTCTCCAAAC	1940	-
tRNA-Glu (UUC)	TCCGATATGGTATAACGGTTAGAACACCTGGC	1593	5′
tRNA-Arg (ACG)	TCAGAGGGTTGCAGGTTCGGGTCCTGTCACGGATGC	1415	3′
tRNA-Asp (GUC)	TTCTCGGTAGTATAGTGGTAAGTATACCCGCCTGTCAC	1160	5′
rRNA	ATATCGAATCGCCATCCAAATCATCTGGTAGGCTCTGCCTGCCC	1034	-
tRNA-Thr (UGU)	GGCCTCGTAGCACAGTGGTAGTGCACTGGT	1010	5′
rRNA	ACACGTCCCTCTCCAAACGAGAGAACATGCATGGGCTGGCATGAGCGG	597	-
snRNA	AAGGCATCGTCGTTTCGACTTTTACTAAGCGACGCAGCCCAAAC	174	-
snoRNA	TAACCGCGGGTAGCACCGTTGTGGAGCACAAAC	15	-

a The type of non-coding RNA from where this small RNA is derived. The triplet inside the parenthesis refers to the anticodon of the tRNA gene.

b The sequence of the small RNA.

c Refers to the number of identical copies of this small RNA that was found in the sequencing data.

d For small RNAs derived from tRNAs this refers to if the small RNA is derived from 3′ or 5′ of a mature tRNA.

A recent study reported the cloning and characterization of tsRNA in the primitive eukaryote *Giardia lamblia* (*G. lamblia*) [38], showing that tsRNA are abundantly expressed during the encystation stage and are ∼46 nt long. Consistent with *T. cruzi* tsRNAs, *G. lamblia* tsRNAs are derived from most tRNA isoacceptors and predominantly from the 3′ arm. In *G. lamblia*, tsRNAs from tRNA^Asp^ and tRNA^Gly^ were the most frequently cloned, which may indicate species or life stage specific isoacceptor preference.

If tsRNA would represent degradation products from tRNA-turnover, it would be expected to find a correlation between the RNA fragment amount and the expression levels of tRNA genes. In the absence of tRNA expression data, we utilized the amino acid usage from the predicted proteome and compared it with the observed tsRNA expression. We found no correlation between the observed tsRNA expression and the amino acid usage (Pearson's correlation, r = −0.05), nor was there a correlation between the genomic copy number of tRNA and tsRNA expression (Pearson's correlation, r = 0.08), suggesting that *T. cruzi* tsRNAs are not random degradation products from tRNA turnover. As we observed a very high expression of tsRNA from certain tRNA isoacceptors (e.g. tRNA^His^, tRNA^Arg^ and tRNA^Thr^), but almost no expression from others (tRNA^Phe^ and tRNA^Asn^), this implies tsRNA are differentially expressed in *T. cruzi*. Furthermore, we performed experimental validation of a few selected tsRNA (Figure S3).

Consistent with previous reports [36], [37], [38], [39], we found that the cleavage site was present within the anticodon loop of mature tRNAs (Figure S2). For shorter tsRNAs, the cleavage site was present in the two other loops, but primarily in the loop of the T-arm. This suggests endonucleolytic cleavage as the responsible mechanism behind tsRNA generation. The precise cleavage supports the idea that tsRNA are generated by a distinct mechanism rather than random degradation. However, as shorter tsRNA were observed, these might require both endonucleolytic cleavage and exonucleolytic trimming in their biogenesis pathway.

We observed tsRNA with and without a CCA 3′ terminus, thus the process of tsRNA formation likely targets both pre-tRNAs and mature tRNAs, and therefore takes place either in the cytosol or nucleus, as only mature tRNAs are imported into the mitochondria [45]. An early study by Zwierzynski et al. reported 3′ CCA activity in nuclear extracts [46], raising questions about the subcellular location of tsRNA biogenesis. The key enzymes involved in tsRNA biogenesis remain to be identified; however, it remains clear that this mechanism is independent of Dicer.

It has been hypothesized that tsRNAs inhibit protein synthesis either by depleting the cellular tRNA pool or by a more intrinsic mechanism involving a protein repression complex [43], albeit there is to date no definite evidence. tsRNAs have been associated with Piwi and Argonaute complexes [47], [48], [49], suggesting that it may guide degradation of target transcripts in RNAi-positive organisms. A recent study reported tsRNAs to guide tRNase Z-mediated cleavage of engineered target sequences and possibly endogenous transcripts [50], which further supports the idea of these species as functional entities.

### Small RNAs derived from other major non-coding RNAs

Small nucleolar RNAs (snoRNAs) are present throughout eukaryotes and guide enzymatic modifications of target RNAs in the nucleolus, and can be subdivided into C/D and H/ACA classes based on sequence motifs. Recently, snoRNA-derived small RNAs (sdRNA) have been reported in animals [37], [51], [52] and in the protozoan *G. lamblia* [53] and are thought to be generated by a Dicer-dependent mechanism [52]. Metazoan sdRNAs are predominantly ∼17–19 nt and ∼30 nt and generated either from the 5′ (C/D type snoRNAs) or 3′ ends (H/ACA type snoRNAs) [52]. In both humans and *G. lamblia* snoRNA-derived small RNAs have been implicated to have miRNA-like functions [53], [54].

We found that 0.26% (1413/528,228) of the total data was represented by snoRNA-derived small RNAs, with a median length of 35 nt, similar for both C/D and H/ACA (Figure 1G, Figure 2). The observed length of sdRNA is different from metazoan sdRNA and both types were found to have similar number of reads (n = 770 and n = 643 reads for C/D and H/ACA snoRNA respectively). We did not observe the positional bias towards the 3′ end which has been reported for mammalian sdRNA, or a specific alignment pattern suggestive of regulated cleavage. These findings suggested that the observed sdRNAs were generated by a different mechanism compared to those found in metazoans, or less interestingly, represent degradation or break-down products.

A total of 0.53% (2839/528,228) reads were derived from small nuclear RNAs (snRNAs) which were distinct from snoRNAs, with a median length of 40 nt. Interestingly, 82.1% (2333/2839) of the snRNA derived small RNAs were from snRNA U4 and U5. Two snRNA-derived reads occurred in a high copy number (∼100 copies).

Small RNAs derived from ribosomal RNA have received less attention but are known to exist [37], [40] and have been reported to increase as a response to oxidative stress. Here, 17.2% (91,206/528,228) of the aligned sequences represented small RNAs derived from ribosomal RNAs (rsRNAs). rsRNAs could be grouped into three different subpopulations based on their length distribution (Figure 1F); one population with an average length of 20 nt, a second population with an average length of 33 nt, and a third longer population with an average length of 46 nt. Complete rRNA genes are not present in the current assembly [2], [3] and it is therefore difficult to conclude if the small RNAs represent degradation products or not. However, the copy number of rRNA-derived small RNAs was highly variable; ranging from 1 (n = 6337 reads) to >100 (n = 117 reads), which suggests a mechanism of non-random degradation.

### Novel transcribed small RNA loci

A total of 1.69% (8964/528,228) of the aligned reads were not derived from known tRNA, rRNA, snRNA, snoRNA or repeats, of which 17.4% (1565/8964) aligned with protein-coding genes and the remaining with intergenic regions (Figure 1HI). In order to find novel ncRNAs, we performed clustering of reads with overlapping alignments (Materials and Methods).

These criteria formed 92 loci, consisting of a total 7805 reads (Table 6, Table S1), of which 13 loci were identified as known non-coding RNAs using homology searches, which have been missed in the present genome annotation. The remaining 79 loci did not fall into known ncRNA classes and had an average length of 54 nt. None of these had homology to any known RNA class in Rfam or GenBank, albeit seven displayed partial sequence similarity with protein-coding genes and pseudo genes. We performed secondary structure prediction [29] of these unknown RNAs; 26 did not fold at all, 35 folded into non-hairpin structures and 18 folded into hair-pin structures according to predictions. Next we compared the 79 candidates to ncRNAs previously reported from comparative genomics [18], but failed to find overlap between the two sets of candidates. This result does not exclude the possibility that the previously reported ncRNA are correct, as only 20% (15/72) was in the size range of our library. Finally we queried our 79 novel ncRNA candidates against other trypanosomatid genomes (*T. brucei*, *T. vivax*, *T. congolense*, *Leishmania* spp.) to test if these sequences are conserved among other trypanosomatids; however, no full length matches were found. These findings suggested that novel RNAs, as identified here, are specific for *T. cruzi* rather than ubiquitous among trypanosomatids.

**Table 6:** Identified novel expressed loci.

Annotation a	Number b	#Reads c
tRNA-Ala gene	1	3328
Unknown RNA	79	3104
Spliced leader RNA	4	807
Signal recognition particle RNA	2	601
tRNA Selenocysteine	1	260
snRNA U5	1	96
rRNA	3	86
H/ACA snoRNA	1	71

a See Table S1 for genomic coordinates.

b Refers to the number of small RNAs determined from clustering of aligned sequence reads.

c Refers to the number of single mapping reads.

The remaining 1159 reads did not pass the criteria for clustering and had a median length of 24 nucleotides. These reads were subsequently queried with BLAST against a trypanosomatid sequence database (Materials and Methods); 335 reads displayed homology to trypanosomatid rRNA genes and 819 with homology to protein-coding genes. For reads with alignment to protein-coding genes we observed no statistical overrepresentation of antisense alignments, and as these did not derive from known ncRNA, the following scenarios are possible; i) small RNAs with homology to protein-coding genes are spurious transcriptional products, or debris from mRNA turnover, without biological significance, ii) small RNAs with homology to protein-coding genes are a result of regulated or non-regulated mRNA turnover with biological significance, iii) small RNAs with homology to protein-coding genes are transcribed from the genome and not derived from mRNA. To address these questions, functional studies will be needed to answer whether these small RNAs are biologically active or debris from the normal cellular turnover.

MicroRNA (miRNA) is a class of regulatory small RNAs that fine tune gene expression in metazoans. One attractive hypothesis is that intracellular parasites utilize the host microRNA pathway to change the cellular environment for its own needs. Partial evidence exists from *Cryptosporidium parvum* and *Toxoplasma gondii* that this may take place [55], [56], [57]. None of the small RNAs showed complete or partial homology when compared with human microRNA sequences from [58]. Next, we performed putative target site prediction of the 819 small RNAs. The putative ‘seed region’ (nt 2–8) was extracted from each of the 819 small RNAs and queried using standalone TargetScan against 23-way UTR alignments. A conserved target site was required to be present in the following 7 genomes; *Homo sapiens*, *Mus musculus*, *Rattus norvegicus*, *Gallus gallus*, *Macaca mulatta*, *Pan troglodytes* and *Canis lupus familiaris*. As a result, a total of 3230 putative target genes were identified. Subsequently, a slimmed gene ontology was used to group the identified genes into a more narrow set of categories. Interestingly, 33% (1063/3230) of the genes grouped into ‘cellular nitrogen compound metabolic process’ (GO:0034641), raising the possibility that parasites may modulate the immune response by interfering with the host production of nitric oxide. Furthermore, ‘immune system process’ (GO:0002376) contained 7.7% (250/3230) of the genes. One hypothesis derived from this bioinformatic prediction is that *T. cruzi* manipulates the host cell environment by secretion of oligonucleotides that mimic human microRNAs.

### Small RNAs derived from repeats

Repeats are an inherent feature of most eukaryotes and have been attributed as an important driving force behind genome evolution [59]. *T. cruzi* have a significant part of its genome devoted to repeats; inactive and active retrotransposons, microsatellites and large gene families, often arranged in tandem. At least two types of non-Long Terminal Repeat (LTR) retrotransposons, designated CZAR and L1, are potentially active in the *T. cruzi* genome [60]. The CZAR element consists of two open reading frames and represent a site-specific retrotransposon that inserts into spliced-leader genes [60]. Small RNAs have been implicated in the protection against retrotransposons in both metazoans and protozoa [22]. However, it is presently unknown how RNAi-negative protozoa, such as *T. cruzi*, protect themselves against the potentially disruptive effects of transposition events. This intriguing question motivated us to look for evidence of small RNAs that target or transcribe from retrotransposons and other repeats.

Initially, the *T. cruzi* genome was searched with RepeatMasker [25] in combination with RepBase [26] to identify all known instances of mobile elements and satellite repeats, which resulted in 13 different types of repetitive elements covering 11–12% of the genome (Table S2). Twenty base pairs flanking each side of a repeat instance was included. To add more confidence to the analysis, we decided to maximize the number of useable reads by including those that go to multiple locations (multi mapping reads). We used a similar approach to what was described in [61], where a particular read was allowed mapping to more than one location, but only to one type of element. Reads going to more than one type of element or outside of repeats were removed. This resulted in a total of 0.13% (782/582,243) of reads from the library that aligned with various repetitive elements (Table S2). This suggests that if any of these small RNAs have a role to inhibit or block transposition events, these are present in a very low amount.

We found that CZAR contained the highest amount of aligned reads (n = 446), despite the fact that this element only covered 0.21% of the genome. Several instances of the CZAR element were found to have reads in the 5′ or 3′ termini, or in the close vicinity. As reads were mostly found to align sense, these may represent initiation fragments from the transcription of these elements, supporting the idea that at least some CZAR elements are actively transcribed in the genome.

The SIRE and TcVIPER have been suggested to represent two classes of dead elements [60]. A low number of reads aligned with TcVIPER (n = 25) and SIRE (n = 2), possibly suggesting that some transcription of these elements might occur despite their inability to transpose.

TcSAT1 is a ∼200 bp satellite repeat and comprises ∼5% of the current draft genome sequence [62]. Conflicting data exist regarding the transcription of TcSAT1, where Northern blot hybridization experiments indicated no transcription, whereas nuclear-run-on assays and microarrays indicated active transcription (see [62] for references). We found 150 reads aligned with this repeat element, which may represent degradation fragments or small RNAs derived from longer transcripts.

Overall, we observed no overrepresentation of antisense reads in any class of repeat elements. However, it is possible that an antisense inhibitory mechanism is present albeit in a very low abundance, which would require deeper sequencing and a more narrow size fraction to be captured. Finally, it is also possible that *T. cruzi* does not use small RNAs to control transposition.

### Validation of selected small RNAs using stem-loop real time PCR

We validated the presence of 12 small RNAs that were found to be abundant in the sequencing data; six tsRNAs (derived from tRNA^Ala^, tRNA^Tyr^, tRNA^Trp^, tRNA^Glu^, tRNA^Asp^ and tRNA^Thr^), four rsRNAs and a repeat-derived small RNA (Figure S3). Validation was performed by Stem-loop Real Time PCR [32], which has previously been used to detect microRNAs and is more sensitive than Northern hybridization [63]. Of the 18 selected small RNAs, 12 could be amplified (Figure S3AB). A tsRNA derived from tRNA^His^ was also detected among our samples (data not shown), however, due to primer dimer formation it could not be properly quantified by real time PCR analysis.

To obtain a measure of abundance, the signal intensity from the real time PCR was normalized using full length rRNA and C/D snoRNA (Materials and Methods). A tsRNA derived from tRNA^Asp^ gave the strongest signal of the tested tsRNAs (Figure S3AC). The other five tsRNAs displayed a similar level of expression as the snoRNA-control used in these experiments. Three tsRNAs (derived from tRNA^Ala^, tRNA^Glu^, tRNA^Asp^) have been detected previously in the *T cruzi* clone Dm28c by Northern hybridization [19], thus indicating their presence in independent strains.

Of the four tested rsRNAs, rsRNA-2 gave the strongest signal (Figure S3BC). Interestingly, several small RNAs derived from non-coding RNAs can be aligned with protein coding genes in the anti-sense direction. For example, rsRNA-3 and rsRNA-4 can be aligned with two distinct protein-coding genes, along with several other putative small RNAs. A similar situation occurs with MASP genes, where small RNAs derived from the repeat element TREZO [64] can be aligned close to the MASP 3′ UTR, which is the most conserved region among these genes [65]. We validated a small RNA derived from the TREZO element, showing that the abundance is similar to that of the snoRNA control (Figure S3). TREZO elements cover ∼1–2% of the genome, exhibit site-specificity for insertions and are transcribed [64], although this is the first report to show they generate such small RNAs. Their putative influence on the MASP family expression needs to be further investigated.

### Conclusions

In this study, we analyzed the short transcriptome of *Trypanosoma cruzi* using unbiased deep sequencing and provided a glimpse into the diversity and abundance of small RNAs in this species. Despite the fact that *T. cruzi* lacks RNA interference, our deep sequencing led to the identification of several new types of small RNAs which have not previously been reported in this important organism. The most common RNA species were small RNAs derived from transfer RNAs, followed by small RNAs derived from ribosomal RNAs. Only 1% of the small RNAs in the library were derived from small nuclear RNAs and small nucleolar RNAs. Our deep sequencing effort confirms that, similarly to other protozoan species and mammalian cell lines, *T. cruzi* accumulates RNA species from tRNA, rRNA as well as snRNA and snoRNA. A selected set of small RNAs was validated using real time PCR and found to be consistently present in different biological samples, although further experimental work will be needed to provide functional insights into the putative roles of some of these small RNAs. Our sequencing data provide a substantial number of follow-up candidates which might be suitable for detailed experiments.

We found no evidence of canonical small non-coding RNAs (i.e. microRNA and siRNA) as often found in metazoans; an expected finding, consistent with the absence of the RNA interference machinery and confirms the results from previous studies showing that canonical microRNAs do not exist in *Trypanosoma cruzi*. About 1.69% of the small RNAs in the library were unknown, and we identified 92 novel expressed loci, of which 79 lacked conserved sequence or structural motifs. However, it should be noted that the small RNAs reported in this study may not reflect the complete repertoire, as certain small RNAs may have a life stage specific expression or otherwise only be expressed under a certain physiological condition.

Further sequencing efforts will be needed to elucidate the complete set of small RNAs and to completely distinguish biologically non-stable intermediates from stable RNAs. Furthermore, it remains to be elucidated whether small RNAs are generated by a distinct mechanism or produced by RNA decay, although the latter does not exclude the possibility that small RNAs have a functional role. Currently we are undertaking deep sequencing of a smaller size fraction to further understand the composition and complexity of the short transcriptome in this peculiar organism.

## Supporting Information

Figure S1 **tsRNAs grouped by tRNA isoacceptor precursor.** Length distributions of tRNA-derived small RNAs per tRNA isoacceptor. The read count is present on the Y-axis and read length (nt) on the X-axis.
(PDF)

Figure S2 **tRNA-derived small RNAs in relation to tRNA secondary structures.** Displays six examples of small RNA derived from tRNA isoacceptors. The small RNA is shown in red. The following tRNA isoacceptors are included; Arg, His, Val, Trp, Gln, Asp. Secondary structure prediction of tRNAs was performed using tRNAscan-SE and visualized using VARNA.
(PDF)

Figure S3 **RNAs validated by stem-loop real-time PCR.** A) and B) show stem-loop RT-PCR products of 6 validated tsRNAs and 4 rsRNAs, respectively. Stem-loop real-time PCR (Chen et al, 2005, NAR) adds an additional 48 bases to the amplified products, resulting in fragments larger than the library sizes. Negative control: no reverse transcriptase (-RT). Positive controls: 5S rRNA and snoRNA. Molecular sizes in base pairs are indicated to the right. C) Stem-loop real-time PCR intensities are shown as relative abundance of validated tsRNA and rsRNA, normalized against 5SrRNA. Graph shows mean values and standard deviation for triplicates of one biological sample. A similar profile was also generated in biological duplicates. snoRNA-C: snoRNA-control. Repeat-1: sense small RNA mapped to the repeat element TcTREZO at MASP 3′UTR multi-locus.
(PDF)

Table S1 Genomic coordinates of novel expressed loci.
(XLS)

Table S2 i) Summary of repetitive elements identified in the *T. cruzi* genome sequence. ii) Table with the number of reads that align within each type of element.
(XLS)

Table S3 **List of primers used for reverse transcriptase and PCR reactions.** Primer sequences are 5′ to 3′. Bold letters on SLRT primers indicate the sequence of the Universal reverse primer used in PCR step. The sequence 3′ of/or after/the dash (-) in both SLRT and F primers, are specific to the respective small RNAs.
(XLS)
