# Supplementary figures and images for: The Short Non-Coding Transcriptome of the Protozoan Parasite Trypanosoma cruzi
Source: PLoS Negl Trop Dis. 2011 Aug 30;5(8):e1283. doi: 10.1371/journal.pntd.0001283 (PMC3166047; doi:10.1371/journal.pntd.0001283)

# tsRNA grouped by tRNA isoacceptor precursor

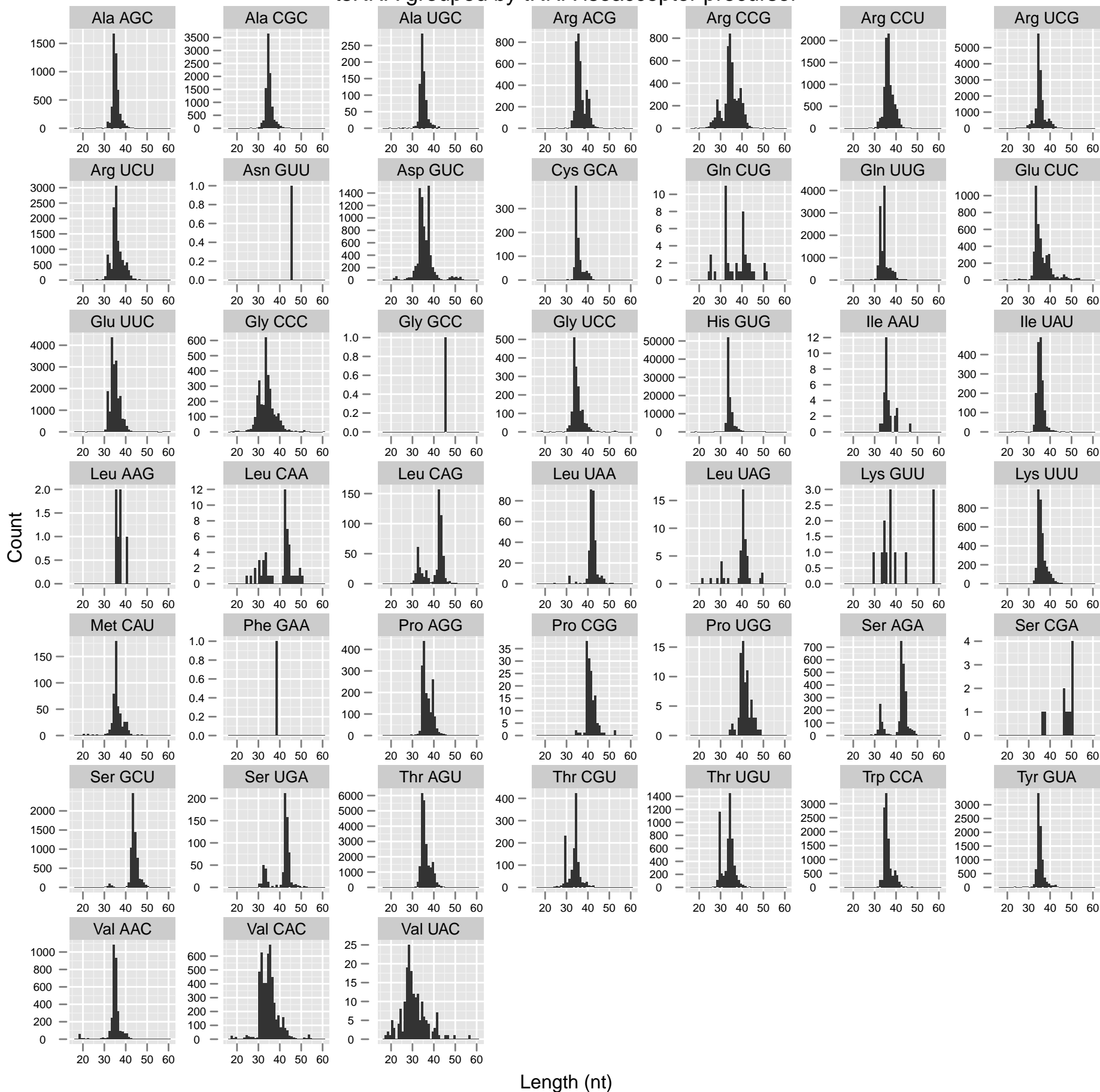

Supplement: Figure S1 — tsRNAs grouped by tRNA isoacceptor precursor. Length distributions of tRNA-derived small RNAs per tRNA isoacceptor. The read count is present on the Y-axis and read length (nt) on the X-axis. (PDF) [file pntd.0001283.s001.pdf]

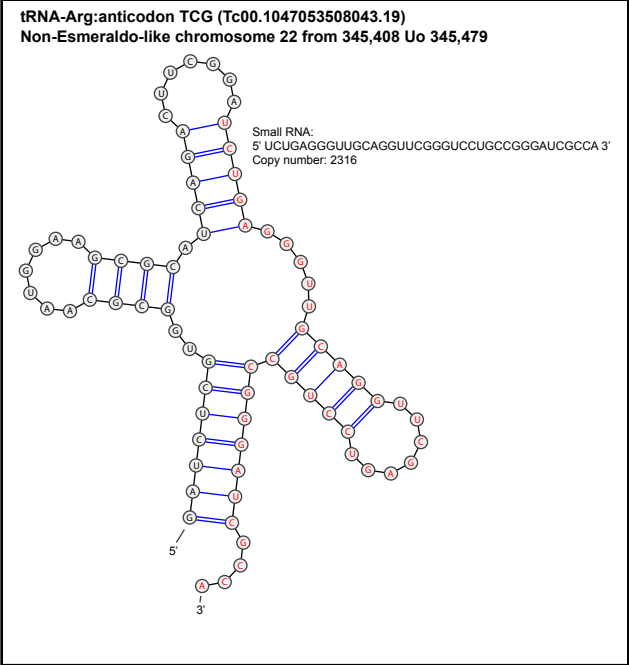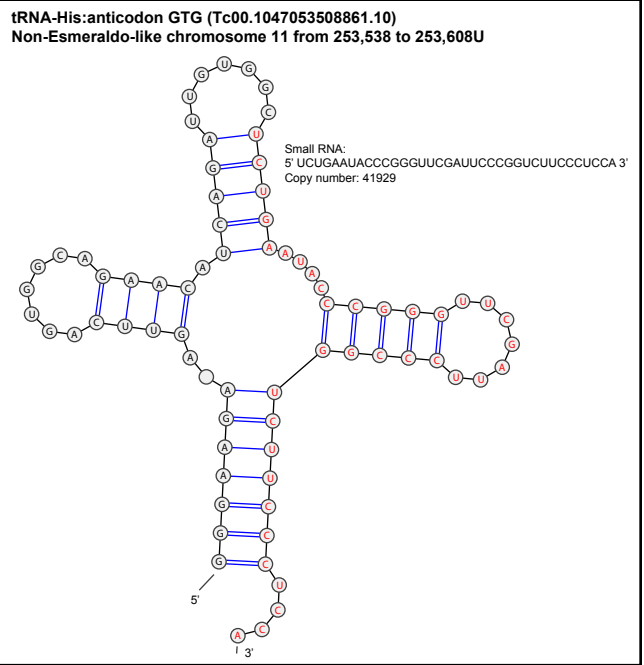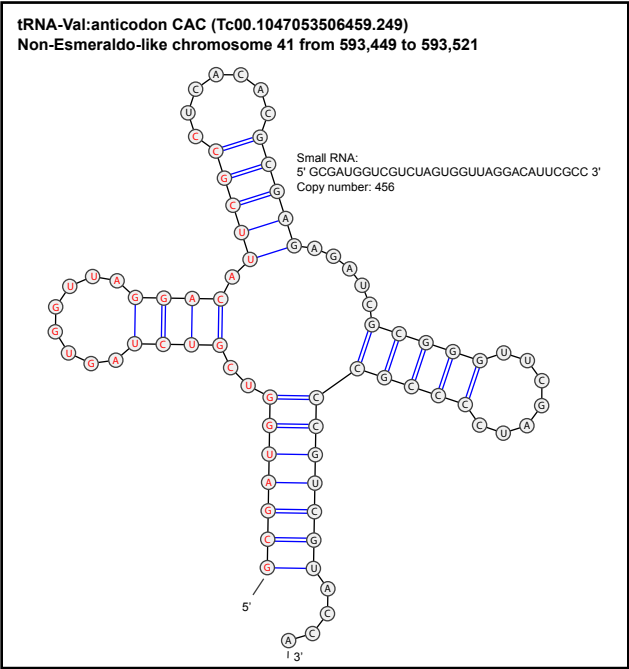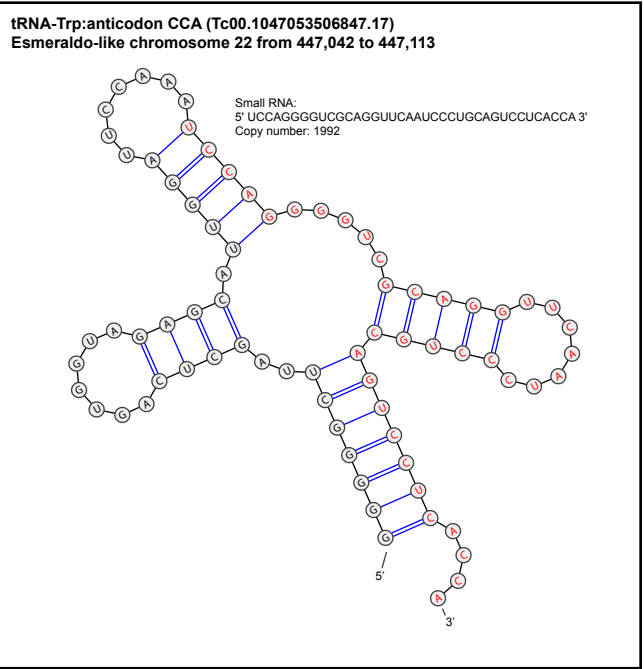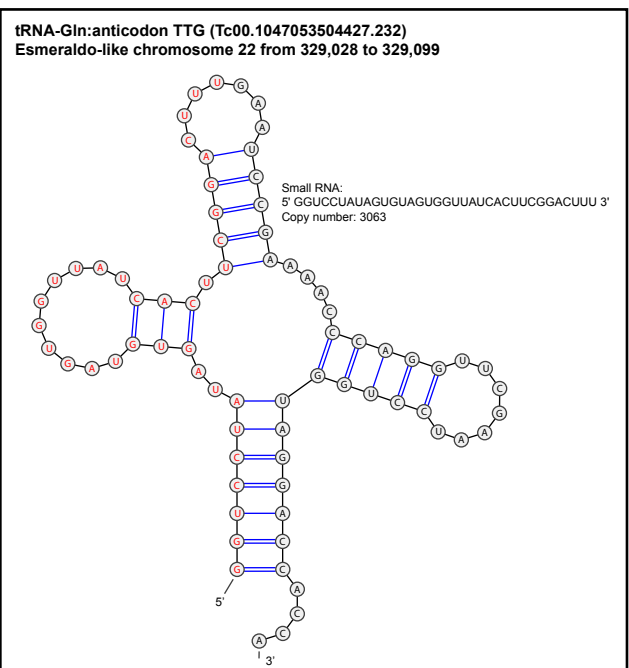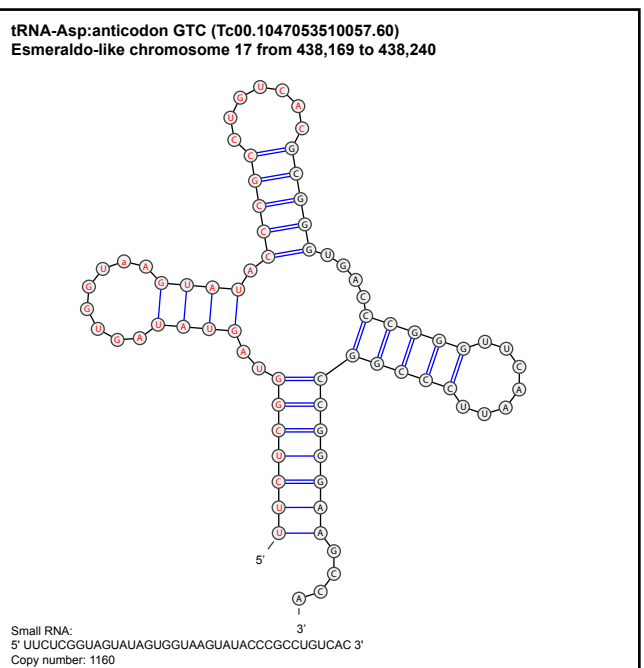

Supplement: Figure S2 — tRNA-derived small RNAs in relation to tRNA secondary structures. Displays six examples of small RNA derived from tRNA isoacceptors. The small RNA is shown in red. The following tRNA isoacceptors are included; Arg, His, Val, Trp, Gln, Asp. Secondary structure prediction of tRNAs was performed using tRNAscan-SE and visualized using VARNA. (PDF) [file pntd.0001283.s002.pdf]

A

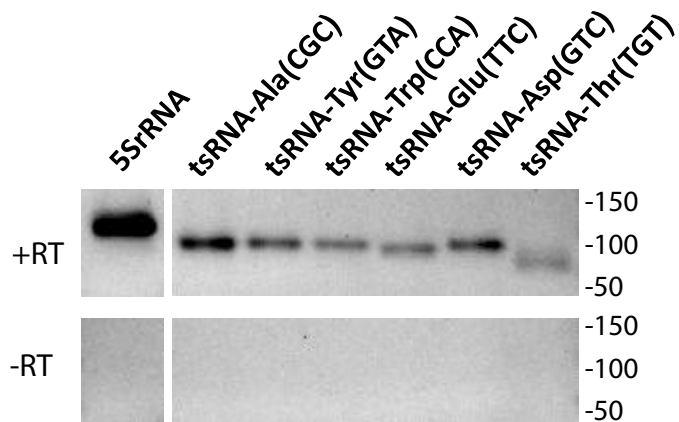

B

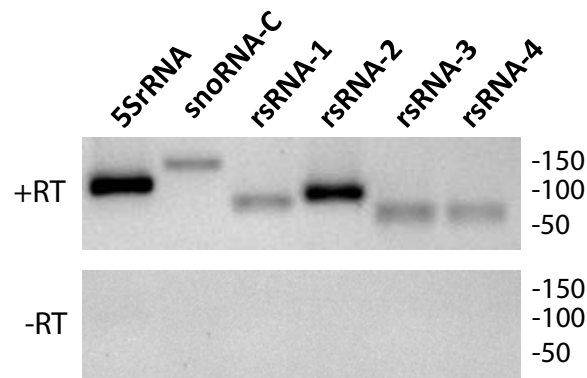

C

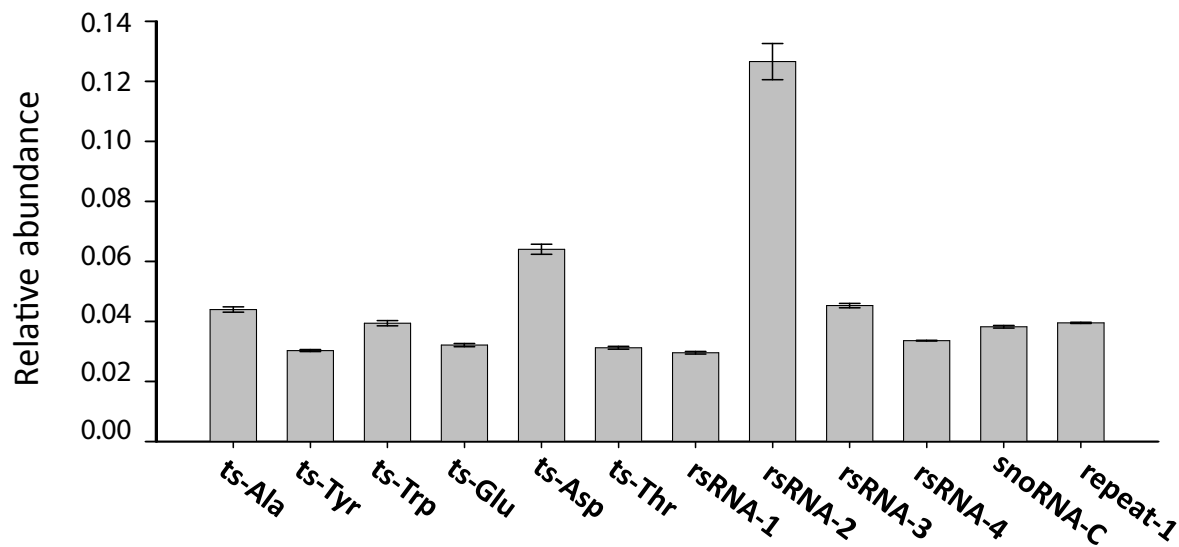

Supplement: Figure S3 — RNAs validated by stem-loop real-time PCR. A) and B) show stem-loop RT-PCR products of 6 validated tsRNAs and 4 rsRNAs, respectively. Stem-loop real-time PCR (Chen et al, 2005, NAR) adds an additional 48 bases to the amplified products, resulting in fragments larger than the library sizes. Negative control: no reverse transcriptase (-RT). Positive controls: 5S rRNA and snoRNA. Molecular sizes in base pairs are indicated to the right. C) Stem-loop real-time PCR intensities are shown as relative abundance of validated tsRNA and rsRNA, normalized against 5SrRNA. Graph shows mean values and standard deviation for triplicates of one biological sample. A similar profile was also generated in biological duplicates. snoRNA-C: snoRNA-control. Repeat-1: sense small RNA mapped to the repeat element TcTREZO at MASP 3′UTR multi-locus. (PDF) [file pntd.0001283.s003.pdf]
